# Thoracic venous congestion caused by thoracic disc herniation

**DOI:** 10.1002/brb3.127

**Published:** 2013-02-17

**Authors:** Eric P Roger, Andrea J Chamczuk, Marygrace C Hagan

**Affiliations:** Department of Neurosurgery, School of Medicine and Biomedical Sciences, University at Buffalo, State University of New YorkBuffalo, New York

**Keywords:** Foix–Alajouanine syndrome, spinal arteriovenous fistula, thoracic disc herniation, venous congestive myelopathy

## Abstract

We present what is to our knowledge the first reported case of thoracic disc herniation leading to venous congestive myelopathy (VCM), which was clinically and radiographically suggestive of Foix–Alajouanine syndrome (angiodysgenetic necrotizing myelopathy). In addition, we review current concepts in evaluating the etiology of VCM and discuss indications for surgery.

## Introduction

Venous congestive myelopathy (VCM) (Matsuo et al. 2008) is classically associated with spinal dural arteriovenous fistula (AVF) and formerly known as Foix–Alajouanine syndrome (FAS) (angiodysgenetic necrotizing myelopathy) (Foix and Alajouanine 1926). Afflicting men in mid-to-late adult years, manifestation consists of progressive paraparesis and sensory dysfunction of lower extremities. Compromise of bowel, bladder, and sexual function frequently follows. Hallmarking the disease are clusters of enlarged and tortuous subarachnoid veins and patchy necrosis of spinal cord tissue. VCM typically represents an irreversibly damaged state resulting from advanced congestive venous hypertension of the spinal cord. Spinal dural AVF is not always detected. Here, we present what is to our knowledge the first reported case of thoracic disc herniation leading to VCM, which was clinically and radiographically suggestive of FAS.

## Case Description

A 67-year-old, right-handed man with a history of hypertension and deep-vein thrombosis presented with a 1-year history of progressive lower extremity paraparesis that began with mid-thoracic pain. Spine magnetic resonance (MR) imaging performed at an outside institution demonstrated T2 hyperintense signal changes within the cord at T9, without evidence of focal cord compression, suggestive of intramedullary thoracic tumor. Lumbar puncture, electromyogram, nerve conduction study, and brain MR imaging were unrevealing. He underwent laminectomy for biopsy; however, upon opening, a small engorged superficial vessel was encountered along the dorsal cord. Suspicion of an arteriovenous malformation (AVM) resulted in abortion of the procedure. The patient was transferred to our institution.

**Figure 1:**
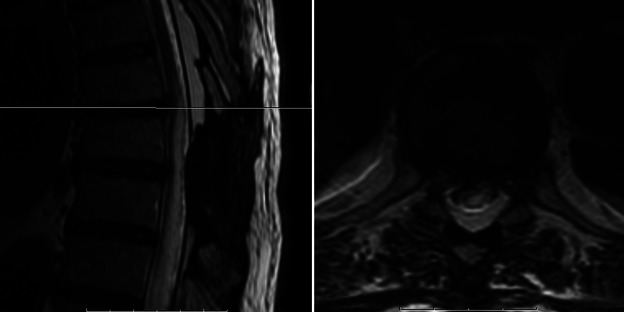
Preoperative focused T2-weighted sagittal (left) and corresponding axial (right) magnetic resonance (MR) images show significant cord edema terminating at the T7–8 disc, where right-sided herniation with cord compression and distortion is noted.

**Figure 2:**
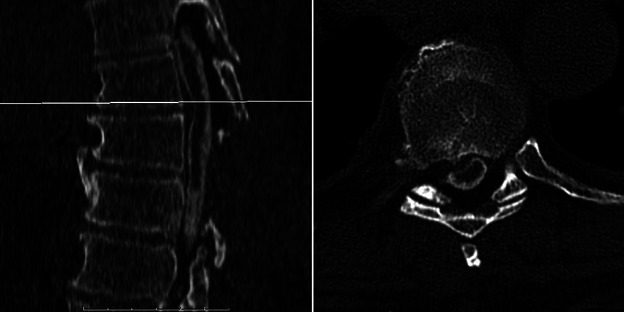
Preoperative focused computed tomographic myelogram, sagittal (left) and corresponding axial (right) views, showing right paracentral subligamentous disk herniation at T7–8 with mass effect on the cord.

On evaluation, the patient demonstrated bilateral paresis, with the right leg more affected than the left, precluding ambulation. Sensory examination demonstrated a spinothalamic sensory level at T6 on the right. Proprioception was unaffected, but neurogenic bladder and bowel had ensued. He had clonus bilaterally. Selective spinal angiography from T4–L1 failed to reveal AVF or AVM. Thoracic and lumbar spine MR imaging was repeated with a 3-tesla magnet and demonstrated severe cord edema versus a syrinx at and below T8, with questionable venous infarction (Fig. 1). A moderate-sized disk herniation with cord distortion was noted at T7–8. Computed tomographic (CT) myelography confirmed cord compression and cord displacement/distortion at T7–8 (Fig. 2); the severe cord edema impressively terminated at the level of the disc herniation (Fig. 1). He was fully investigated by the neurology service and evaluated for other causes of myelopathy; all these results (including cerebrospinal fluid and blood analyses) were negative. His case was presented at spine conference at our institution, with a consensus toward surgical intervention**.**

**Figure 3:**
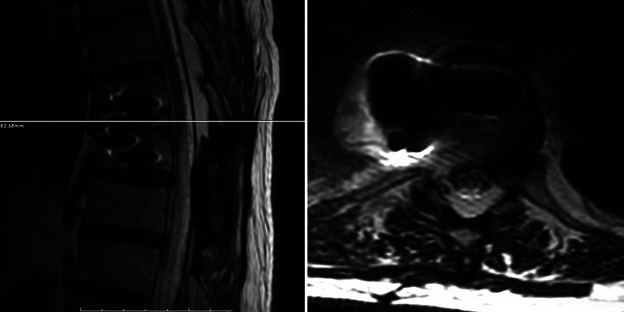
Postoperative focused T2-weighted sagittal (left) and corresponding axial (right) magnetic resonance (MR) images show thorough decompression of the cord.

The patient underwent right transthoracic thoracotomy for T7–8 discectomy with rib autograft interbody fusion with instrumentation. Intraoperatively, no tumor or vascular malformation was identified, but a large draining vein was noted along the dorsal dural surface of the cord. At the time of decompression, the dura was noted to be concavely deformed. After thorough decompression, the dura had completely regained a normal convex shape. Interbody fusion with anterior plating was used in the face of the previous laminectomy. Postoperative thoracic MR imaging confirmed thorough cord decompression (Fig. 3).

The patient convalesced and began ambulating with use of a walker. He regained some element of sphincter control. At the 1-year follow-up office visit, he was ambulating well with the assistance of a walker.

## Discussion

The cause of FAS has been attributed to infection, thrombophlebitis, and spinal AVM or AVF (Matsuo et al. 2008). Scattered reports of venous infarction of the spinal cord have been associated with decompression sickness, gliosarcoma, orchiectomy, pulmonary emboli, furunculosis, leukemia, polycythemia, and thrombosis of leg veins (Srigley et al. 1981; Clarke and Cumming 1987; Niino et al. 1999). In classic FAS, venous drainage of the AVF is to the coronal venous plexus lying posterolaterally over the cord's surface (Renowden and Molyneux 1993). Elevation of venous pressure occurs; the absence of valves promotes transmission of high pressure to the radial intramedullary vein and cord parenchyma, causing myelopathy. From a pathophysiologic perspective, VCM represents irreversibly progressive disease due to damage of the spinal cord parenchyma caused by venous congestion. Unlike cases of hemorrhagic or embolic spinal venous infarction, which typically present with symptoms of sudden onset of low back pain and progress rapidly, VCM carries more insidious onset and protracted course. Although patients uniformly present with paraparesis or sensory disturbance, the pattern of distribution and progression of VCM can be heterogeneous, often precluding prompt diagnosis.

Imaging characteristics compound the diagnostic evasiveness of VCM. Typified by an intramedullary lesion with high signal intensity on T2-weighted imaging, flow voids of tortuous vessels can be seen on the dorsal surface of the spinal cord. The increased T2 signal reflects increased water content of necrotic tissue and the proliferated vessels in VCM. MR imaging can also show mass effect with cord swelling, iso- or hypointense T1 signal changes and parenchymal enhancement with contrast, making it difficult to distinguish VCM from spinal infarction, transverse myelitis, multiple sclerosis (Scolding 2001), or intramedullary tumor (Rodriguez et al. 2005). Not uncommonly, MR findings of VCM with spinal cord enlargement/enhancement are so suggestive of tumor that they prompt biopsy (Rodriguez et al. 2005), as in this case (biopsy obtained at an outside hospital was negative).

It is not inconceivable that a vascular malformation undergoes spontaneous thrombosis (Renowden and Molyneux 1993). Although myelography can demonstrate dilated tortuous coronal venous plexus as serpentine filling defects, in reported cases of spontaneous thrombosis, the cause of abnormal veins visualized by myelography cannot be demonstrated by angiography (Wrobel et al. 1988; Renowden and Molyneux 1993). In the present case, myelography failed to demonstrate any filling defects and spinal angiography was likewise benign. Therefore, spinal dural AVF was unlikely the cause of VCM.

Certainly, it is possible that the venous congestion (proven venous dilatation at the time of the initial laminectomy and a negative spinal angiogram) could have been due to another unknown cause, with cord edema terminating at the level of compression from the disc, incidentally; the patient may have improved neurologically simply due to the natural course of the disease. Alternatively, cord compression from thoracic disc herniation may have led to venous engorgement of the dorsal medullary vein with impaired venous outflow and VCM as an endpoint. This theory is further supported by the abrupt cessation of function and cord signal changes – evident in the MR images – at the disc herniation level, absence of other pathological conditions (AVF, AVM, tumor), and improvement of neurological function after decompression at the disc herniation.

Treatment of thoracic disc herniations is always guided by correlation of radiological and clinical findings. Imaging evidence of cord contact, indentation, or frank cord compression with or without myelomalacia must be corroborated with clinical findings of myelopathy. Age and comorbidities must also be taken into account, given that many thoracic disc herniations with cord compression may require an extensive anterior transthoracic approach. Focal, “significant” cord compression, preferably with myelomalacia, is often an indication for surgical intervention in patients with myelopathic impairment. In this case, the main radiographic finding was the impressive amount of cord edema spanning multiple levels. Various etiologies were suggested and investigated to no avail. In the absence of other explainable causes, and in the face of a patient clearly deteriorating clinically, it seemed reasonable to consider addressing the compressive associated thoracic disc herniation.

## Conclusions

VCM remains an enigmatic, debilitating endpoint associated with a variety of established underlying causes. In the setting of clinical and radiographic evidence of VCM, pursuit of vascular malformation remains imperative. In the absence of demonstrable AVF, alternative underlying etiologies for VCM must be expeditiously sought and corrected to prevent frank spinal infarction from untreated venous congestion. We propose that an associated disk herniation with mass effect may be a rare but treatable cause of VCM. Further research and documented cases would be necessary to validate this theory of disc-induced VCM.
